# Regulatory BC1 RNA and the Fragile X Mental Retardation Protein: Convergent Functionality in Brain

**DOI:** 10.1371/journal.pone.0015509

**Published:** 2010-11-23

**Authors:** Jun Zhong, Shih-Chieh Chuang, Riccardo Bianchi, Wangfa Zhao, Geet Paul, Punam Thakkar, David Liu, André A. Fenton, Robert K. S. Wong, Henri Tiedge

**Affiliations:** 1 Department of Physiology and Pharmacology, The Robert F. Furchgott Center for Neural and Behavioral Science, State University of New York Health Science Center at Brooklyn, Brooklyn, New York, United States of America; 2 Program in Neural and Behavioral Science, State University of New York Health Science Center at Brooklyn, Brooklyn, New York, United States of America; 3 Department of Neurology, State University of New York Health Science Center at Brooklyn, Brooklyn, New York, United States of America; INSERM U901, France

## Abstract

**Background:**

BC RNAs and the fragile X mental retardation protein (FMRP) are translational repressors that have been implicated in the control of local protein synthesis at the synapse. Work with BC1 and Fmr1 animal models has revealed that phenotypical consequences resulting from the absence of either BC1 RNA or FMRP are remarkably similar. To establish functional interactions between BC1 RNA and FMRP is important for our understanding of how local protein synthesis regulates neuronal excitability.

**Methodology/Principal Findings:**

We generated BC1−/− Fmr1−/− double knockout (dKO) mice. We examined such animals, lacking both BC1 RNA and FMRP, in comparison with single knockout (sKO) animals lacking either one repressor. Analysis of neural phenotypical output revealed that at least three attributes of brain functionality are subject to control by both BC1 RNA and FMRP: neuronal network excitability, epileptogenesis, and place learning. The severity of CA3 pyramidal cell hyperexcitability was significantly higher in BC1−/− Fmr1−/− dKO preparations than in the respective sKO preparations, as was seizure susceptibility of BC1−/− Fmr1−/− dKO animals in response to auditory stimulation. In place learning, BC1−/− Fmr1−/− dKO animals were severely impaired, in contrast to BC1−/− or Fmr1−/− sKO animals which exhibited only mild deficits.

**Conclusions/Significance:**

Our data indicate that BC1 RNA and FMRP operate in sequential-independent fashion. They suggest that the molecular interplay between two translational repressors directly impacts brain functionality.

## Introduction

A key determinant in the experience-dependent interpretation of genetic information is provided by local translational control of gene expression at neuronal synapses. The regulated translation of select mRNAs at synaptic sites is now recognized as a core mechanism in the long-term modulation of neuronal interactions [1]–[4]. Several types of translational regulators have been identified in neurons, including the fragile X mental retardation protein (FMRP) [5]–[7] and regulatory BC RNAs [8], [9].

Functional absence of FMRP gives rise to the fragile X syndrome (FXS) [10], a common inherited form of mental retardation that is characterized by cognitive impairments, behavioral abnormalities and, in subpopulations of FXS patients, by epileptic and/or autistic phenotypes [11]. FMRP is an RNA-binding protein that interacts with a subset of neuronal RNAs [12]–[17]. The protein operates as a translational repressor [17]–[19], most likely via association with neuronal polyribosomes [20]–[23] [but see 19], [24].

Regulatory BC RNAs repress translation at the initiation level [25]–[28]. Absence of dendritic BC1 RNA results in neuronal hyperexcitability and epileptogenesis [29]. The apparent phenotypical commonalities between impaired BC1 RNA and FMRP translational control [see 29,30,31] prompt the question whether the two systems are functionally intersecting. Is the overlapping phenotypical output the result of pathway modulations that are implemented sequentially (and thus likely in independent fashion) or concomitantly (and thus possibly in mutually interdependent fashion)? Answers to these questions will be directly relevant to our understanding of the molecular basis of FXS and associated disorders. Therefore, in the present work, we undertook a functional dissection of FMRP and BC1 RNA translational repression pathway interactions using Fmr1−/−, BC1−/−, and BC1−/− Fmr1−/− animal model systems.

## Results

### Exacerbated Hyperexcitability of the CA3 Neuronal Network

We examined synaptic excitability and epileptogenic susceptibility using electrophysiological approaches. Intracellular recordings were performed in CA3 glutamatergic principal neurons of hippocampal slice preparations from BC1−/−, Fmr1−/−, BC1−/− Fmr1−/−, and WT animals. Epileptiform discharges were elicited by application of bicuculline, a GABA_A_ receptor antagonist [31]. This disinhibition causes short (<1.5 s) synchronized discharges that are stable over the recording period in WT animals; they result in elevated synaptic release of glutamate from principal neurons [31].

**Figure 1:**
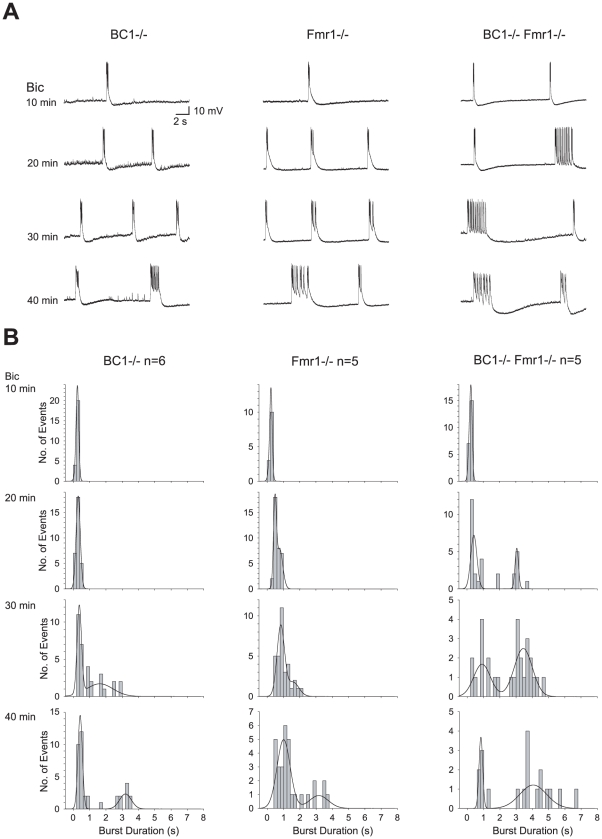
Concurrent absence of BC1 RNA and FMRP gives rise to exacerbated synaptic hyperexcitability of CA3 pyramidal cells. (**A**) Prolonged epileptiform discharges induced by bicuculline (Bic) in hippocampal slices occurred earlier (20 min) in BC1−/− Fmr1−/− slice preparations (right column) than in BC1−/− (left column) or Fmr1−/− (middle column) preparations. (**B**) Summary frequency histograms from each of the three groups of preparations (n, number of slices; one slice per animal) with second-order Gaussian function fits. The appearance of a second distinct peak of burst duration distribution indicates burst prolongation. Bicuculline-elicited burst prolongation occurred in all the three groups but was most prominent in BC1−/− Fmr1−/− preparations, evidenced as follows. (i) Between 10 and 20 min of bicuculline, burst durations significantly increased in the BC1−/− Fmr1−/− group (10 min: 0.213±0.008 s; n = 22; 20 min: 1.316±0.228 s; n = 29; two-way ANOVA followed by Newman-Keuls post-hoc test: P<0.01), whereas burst durations did not change in the BC1−/− (10 min: 0.216±0.005 s; n = 24; 20 min: 0.287±0.016 s; n = 30) or Fmr1−/− (10 min: 0.230±0.013 s; n = 13; 20 min: 0.616±0.026 s; n = 35) groups. (ii) Beginning at 20 min of bicuculline, burst durations were significantly longer in BC1−/− Fmr1−/− slices than in BC1−/− or in Fmr1−/− slices (P<0.01 in all cases). At 40 min bicuculline, the mean duration of long bursts was greater in BC1−/− Fmr1−/− (4.413±0.279 s; n = 13) than in BC1−/− (2.992±0.164 s; n = 11; P<0.001) or in Fmr1−/− slices (2.705±0.264 s; n = 9; P<0.001). (iii) The relative frequency of long bursts (as percentage of total number of bursts) was significantly higher in BC1−/− Fmr1−/− (Bic 30 min: 67.9%; Bic 40 min: 68.4%) than in BC1−/− (Bic 30 min: 21.9%; χ^2^ test, P<0.001; Bic 40 min: 29.7%; P<0.01) or in Fmr1−/− preparations (Bic 30 min: 15.1%; P<0.001; Bic 40 min: 29.0%; P<0.01).

In all three mutant preparations, but not in WT preparations, such short discharges over time transformed into recurrent prolonged synchronized discharges (duration>1.5 s) that are similar to ictal events in epilepsy [Fig. 1; see also 29,31]. This transition of discharge duration from a unimodal phase (short bursts only) to a bimodal phase (long bursts in addition to short bursts) was significantly accelerated in hippocampal slices prepared from BC1−/− Fmr1−/− dKO animals. Prolonged discharges appeared after only 20 min in dKO preparations (Fig. 1A and 1B, right columns), whereas they did not begin to occur until after 30–40 min in sKO preparations (Fig. 1, left and center columns). In addition, at time points 20 min or more after bicuculline application, epileptiform discharges in BC1−/− Fmr1−/− dKO slices were significantly longer (Fig. 1B, 20 and 40 min) and occurred more frequently (Fig. 1B, right column) than in either type of sKO slices. Thus, prolonged discharges (i) appeared earlier, (ii) were of longer average duration, and (iii) occurred at a higher relative frequency in BC1−/− Fmr1−/− dKO preparations than in either BC1−/− or Fmr1−/− sKO preparations. The data indicate that the concurrent absence of BC1 RNA and FMRP precipitates a significantly higher level of neuronal hyperexcitability than the absence of one repressor alone.

### Heightened Epileptogenic Susceptibility

**Figure 2:**
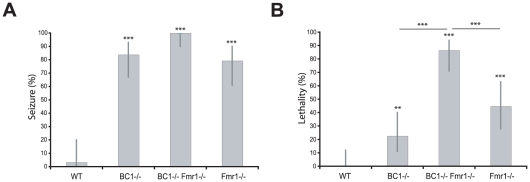
Severe epileptogenic susceptibility of BC1−/− Fmr1−/− dKO animals results in high lethality. (**A**) Lack of BC1 RNA, FMRP, or both, significantly increased propensity for audiogenic seizures (Generalized Linear Model, followed by post-hoc tests of pairs of groups using exact logistic regression stratified by litter; P<0.0001 for each group when compared to WT). (**B**) Rate of lethality resulting from audiogenic seizures was significantly higher in BC1−/− Fmr1−/− animals than in BC1−/− animals (P<0.0001) or in Fmr1−/− animals (P = 0.0007). All mutant animals had a significantly higher audiogenic lethality rate compared with WT (BC1−/− Fmr1−/−, P<0.0001; BC1−/−, P = 0.008; Fmr1−/−, P<0.0001). BC1−/− animals did not significantly differ from Fmr1−/− animals in audiogenic lethality (P = 0.0719). Error bars represent 95% confidence intervals. WT, n = 30; BC1−/−, n = 31; BC1−/− Fmr1−/−, n = 37; Fmr1−/−, n = 29.

To investigate epileptogenic vulnerability in vivo, WT animals and BC1−/−, Fmr1−/−, and BC1−/− Fmr1−/− mutant animals were subjected to auditory stimulation. When exposed to a 120 dB sound, animals of the three mutant mouse strains, but not WT animals, typically initiated excessive motor activity in the form of wild, uncontrolled running and jumping, as has previously been described for BC1−/− and Fmr1−/− sKO animals [29], [32], [33]. Such wild running was followed, within less than 2 minutes, by generalized tonic-clonic seizures (Fig. 2; Movie S1). The percentage of animals undergoing convulsive seizures was high for all three mutant strains (>75%; Fig. 2A). However, while most sKO animals recovered from such seizures within less than 2 minutes, a remarkably high percentage of BC1−/− Fmr1−/− dKO animals died while undergoing seizures (>86% lethality within <90 s after onset of alarm) (Fig. 2B). Thus, BC1-/− Fmr1−/− dKO animals are acutely susceptible to audiogenic epileptogenesis, and epileptic lethality is significantly increased in comparison to BC1−/− or Fmr1−/− sKO animals (Fig. 2).

These results are corroborated by data indicating that initial epileptogenic responses (uncontrolled running) were triggered faster in BC1−/− Fmr1−/− dKO animals than in either BC1−/− animals or Fmr1−/− animals. The percentages of animals in uncontrolled running at 10 s, 15 s, and 20 s after start of the alarm were significantly higher in the BC1−/− Fmr1−/− group than in either the BC1−/− or the Fmr1−/− group (Fig. S1).

The combined results indicate that concomitant absence of BC1 RNA and FMRP results in severely heightened susceptibility to hyperexcitability and epileptogenesis, in comparison with animal models that lack only one type of translational repressor.

### Impaired Place Learning

**Figure 3:**
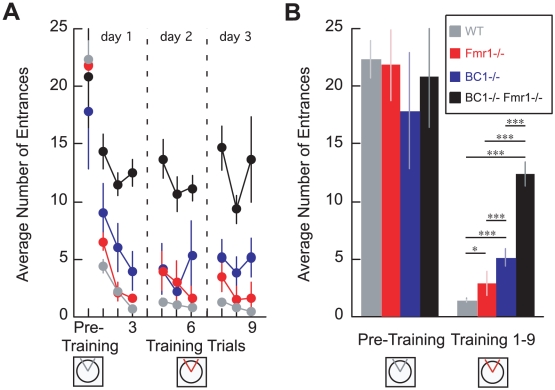
Place learning is mildly impaired in BC1−/− and Fmr1−/− sKO mice but severely impaired in BC1−/− Fmr1−/− dKO mice. (**A**) Exploration, measured as the number of entrances into a 60° zone (gray sector on the circle in the schematic), did not differ amongst the groups on the pre-training trial when the shock was off (F_3,26_ = 0.38; P = 0.8). When the shock was turned on (red sector), all groups except the dKO learned to avoid the shock zone. The effects of genotype and trial across place training were significant (genotype: F_3,234_ = 141.81; P = 10^−52^; F_8,234_ = 4.96; P = 10^−5^) and the interaction was not (F_24,234_ = 0.70; P = 0.85). Newman-Keuls post-hoc tests confirmed that the overall number of entrances significantly differed between all groups (WT<Fmr1−/−<BC1−/−<dKO). The failure of the dKO to learn persisted across 3 days of training (3 trials/day) and this was confirmed by 1-way ANOVA on the factor trial (F_8,45_ = 1.02; P = 0.43). (**B**) Summary of behavior on the pre-training and active avoidance trials illustrates the significant group differences during place learning. Error bars indicate S.E.M., *P<0.05, ***P<0.001. BC1−/−, n = 6; Fmr1−/−, n = 6; dKO, n = 6; WT, n = 12.

We used an active place avoidance paradigm [34] to assess place learning in sKO and dKO animals. As shown in Fig. 3A, WT animals quickly learned to avoid entering a rotating shock zone, reaching their performance asymptote by the third trial on the first day of training. Learning in BC1−/− and Fmr1−/− sKO animals was also robust but retarded in comparison to the WT animals. However, in clear contrast to learning in sKO animals, active place avoidance in dKO animals did not improve at all, even over 3 days of training (Fig. 3).

Thus, while place learning was preserved although retarded in both groups of sKO animals, a severe learning deficit was apparent in dKO animals.

## Discussion

We used three phenotypical readouts -— neuronal network excitability, epileptogenesis, and place learning -— to evaluate the relative contributions of two translational repressors, BC1 RNA and FMRP, to brain function. We report that in all three cases, phenotypical deficiencies were significantly exacerbated in BC1−/− Fmr1−/− dKO animals, relative to BC1−/− or Fmr1−/− sKO animals. As will be discussed in the following, these results have critical implications concerning the mode of functional interactions between the two repressors, BC1 RNA and FMRP.

Neuronal excitability was examined in hippocampal CA3 pyramidal cells. Previous work has shown that in BC1−/− and Fmr1−/− preparations, synaptic glutamate release elicits ictal-like prolonged epileptiform discharges [29], [31]. We now observe that the severity of such hyperexcitability is significantly heightened in BC1−/− Fmr1−/− dKO preparations. This phenotypical exacerbation in the absence of two translational repressors was mirrored in aggravated epileptogenesis in vivo. Sensitivity to audiogenic seizures, as previously described for BC1−/− and Fmr1−/− animals [29], [32], [33] was also observed in BC1−/− Fmr1−/− dKO animals. However, in contrast to sKO animals in which lethality from audiogenic seizures is in the range of 20–30%, audiogenic lethality in BC1−/− Fmr1−/− dKO animals was found approaching 90%. It appears that absence of the translational repressors BC1 RNA and FMRP contribute in modular fashion to phenotypical impairments.

In the active place avoidance paradigm, BC1−/− and Fmr1−/− sKO animals exhibited only mild place learning deficits, results that are in agreement with previous observations that such animals showed no or only mild learning impairments in the Morris water maze and other maze tasks [35]–[37]. In contrast, BC1−/− Fmr1−/− dKO animals were severely impaired in their place learning ability. We note that the active place avoidance task goes beyond testing spatial and navigation ability [38] because optimal performance requires the mouse to segregate the useful spatial information from the stationary spatial frame from the irrelevant spatial information from the rotating spatial frame. Indeed, the ability to segregate spatial information has been dissociated from the ability to form spatial associations [39]. These properties may make the active place avoidance task exquisitely sensitive to even mild hippocampal dysfunction [40], [41], certainly more than the water maze [39], [42], and possibly more than other place learning tasks. In summary, data obtained in three readouts of brain function indicate that concurrent lack of both BC1 RNA and FMRP significantly compounds the phenotypical consequences that are observed in the absence of only one repressor.

**Figure 4:**
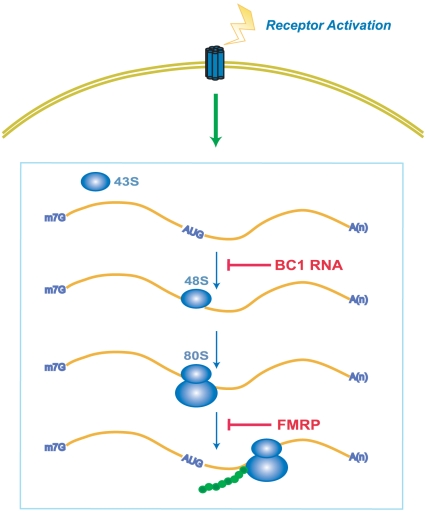
BC1 RNA and FMRP act as repressors on activity-stimulated translation. In this model, a balance of power is maintained in the postsynaptic translation pathway by the functional interplay between two opposing forces: (i) the stimulatory consequences of receptor activation, and (ii) translational repression by BC1 RNA and FMRP. It is suggested that BC1 RNA and FMRP, operating in series in the same translational pathway, target overlapping but non-identical sets of mRNAs. We posit that multiple repressors are needed at the synapse to ensure adequate stimulation-repression homeostasis and to allow for differential derepression options.

The results presented here indicate that the repressors BC1 RNA and FMRP operate sequentially in the translation pathway (Fig. 4). In this scenario, the repressors act in series and independently of each other. Therefore, lack of one repressor will leave the respective other repressor unaffected and functional, and phenotypical output will be less severely impacted than in the case of concurrent absence of both repressors. This model is also compatible with recent results showing that defects of striatal mGluR5-endocannabinoid signaling were significantly exacerbated in BC1−/− Fmr1−/− dKO preparations [43] [but see 44]. Conversely, our results are not compatible with a model in which BC1 RNA and FMRP operate in interdependent fashion [24] as in this case, lack of both repressors should precipitate the same phenotypical consequences as lack of either one repressor. We conclude that the mode of action of BC1 RNA and FMRP is sequential-independent.

Protein-synthetic capacity in synapto-dendritic domains allows a neuron to respond to external stimuli in an input-specific, experience-dependent manner [2], [45]. However, such gains in neuronal plastic responsiveness come at a price: a protein synthetic machinery that, unless properly controlled, may engage in premature or inappropriate translation of locally available mRNAs, resulting in neuronal hyperexcitability. We suggest that effective local translational repression is vital for a neuron to ensure that proteins are only synthesized when and where needed.

BC RNAs and FMRP represent two types of neuronal translational repressors. Previous work has established a balance of power between receptor activation on one hand and translational repression by BC1 RNA and FMRP on the other [7], [29], [46]. Translational repressors thus operate as “brakes” that counteract receptor-mediated translational stimulation (Fig. 4), establishing a stimulation-repression balance that controls translational output in accordance with the physiological status of the synapse. Such brakes have been in place early during phylogenetic brain development as an FMRP ortholog exists in Drosophila [47]. In contrast, dendritic BC RNAs are mammalian-specific, with BC1 RNA restricted to rodents (Rodentia), BC200 RNA restricted to simian primates (Anthropoidea) [48], [49]. It is therefore likely that these RNAs were independently recruited into their current repression function at a later time in mammalian evolution during which increasing brain complexity required more stringent translational control mechanisms. Multiple, mutually independent translational control mechanisms in neurons may also allow for more discrete regulation by upstream signals. The evolution of neuronal RNA-coding genes thus appears to be linked to increasing nervous system complexity in eukaryotes [50], [51].

## Materials and Methods

### Animals

Work with vertebrate animals was in accordance with the Public Health Service Policy on Humane Care and Use of Laboratory Animals and was approved by the State University of New York Brooklyn Institutional Animal Care and Use Committee (Institutional Assurance Number A3260-01, Protocol Number 10-074-09).

BC1−/− mice (lines 13 and 15) were established from independent mutant ES cell lines [52] and used as described [53]. Both lines have a mixed C57BL6/sv129 background. Lines 13 and 15 were used (with equivalent results) for experiments shown in Figs. 1 and 2, line 15 was used for experiments shown in Fig. 3. Fmr1−/− mice carrying the Fmr1^tm1Cgr^ allele were obtained from Jackson Laboratories (Bar Harbor, ME), and have a mixed C57BL6/FVB background. BC1−/− mice were crossed with Fmr1−/− mice to generate BC1−/− Fmr1−/− mice which have a mixed C57BL6/FVB/sv129 background. We used animals at 18–21 days of age except for place learning tasks in which animals at 2–4 months were used.

### Hippocampal Slice Preparations and Electrophysiological Recordings

Transverse hippocampal slices (400 µm) were prepared as described [54]. Slices were allowed to recover from the isolation procedure for at least 1.5 h. Intracellular recordings were carried out in CA3 pyramidal cells using an Axoclamp 2A amplifier (Molecular Devices, Palo Alto, CA) as described [29].

### Auditory Stimulation

Epileptogenic susceptibility to auditory stimuli was tested as described [29]. Animals were subjected to auditory stimulation for 15 minutes. Video recordings were analyzed by a person who was not informed of the animals' genotypes. Recorded parameters included: time to onset of uncontrolled running, time to onset of seizure, percentage of animals undergoing seizures, and lethality.

### Place Learning

We used an active place avoidance paradigm to examine place learning [34]. All animals were trained in a task that requires intact hippocampal function for learning, consolidation and recall [34] as well as hippocampal LTP maintenance by persistent activation of PKMζ for long-term memory storage [42], [55]. The mice were habituated to a rotating arena during a 10-min pre-training session. Active avoidance training began on the following trials by activating a stationary 60° sector as a shock zone.

Data Analysis and Statistical Evaluation were performed as described [29].

## Supporting Information

Figure S1 **Initial epileptogenic responses were triggered significantly faster in BC1−/− Fmr1−/− dKO animals.** Percentages of animals in uncontrolled running after 10 s, 15 s, and 20 s were significantly higher in the BC1−/− Fmr1−/− group than in either the BC1−/− or the Fmr1−/− group (Generalized Linear Model, followed by post-hoc tests of pairs of groups using exact logistic regression stratified by litter, P<0.0001 for 10 s, 15 s, and 20 s groups). The BC1−/− and the Fmr1−/− groups did not significantly differ from each other. Error bars represent 95% confidence intervals. (EPS)

Movie S1 (MP4)
